# Simple derivation of skeletal muscle from human pluripotent stem cells using temperature‐sensitive Sendai virus vector

**DOI:** 10.1111/jcmm.16899

**Published:** 2021-09-12

**Authors:** Ghee Wan Tan, Takayuki Kondo, Keiko Imamura, Mika Suga, Takako Enami, Ayako Nagahashi, Kayoko Tsukita, Ikuyo Inoue, Jitsutaro Kawaguchi, Tsugumine Shu, Haruhisa Inoue

**Affiliations:** ^1^ Center for iPS Cell Research and Application (CiRA) Kyoto University Kyoto Japan; ^2^ iPSC‐based Drug Discovery and Development Team RIKEN BioResource Research Center (BRC) Kyoto Japan; ^3^ Medical‐risk Avoidance based on iPS Cells Team RIKEN Center for Advanced Intelligence Project (AIP) Kyoto Japan; ^4^ R&D Center ID Pharma Co., Ltd Tsukuba Japan

**Keywords:** differentiation method, disease modelling, high temperature treatment, human embryonic stem cells, human‐induced pluripotent stem cells, Myod1, Sendai virus, skeletal muscle

## Abstract

Human pluripotent stem cells have the potential to differentiate into various cell types including skeletal muscles (SkM), and they are applied to regenerative medicine or in vitro modelling for intractable diseases. A simple differentiation method is required for SkM cells to accelerate neuromuscular disease studies. Here, we established a simple method to convert human pluripotent stem cells into SkM cells by using temperature‐sensitive Sendai virus (SeV) vector encoding myoblast determination protein 1 (SeV‐Myod1), a myogenic master transcription factor. SeV‐Myod1 treatment converted human embryonic stem cells (ESCs) into SkM cells, which expressed SkM markers including myosin heavy chain (MHC). We then removed the SeV vector by temporal treatment at a high temperature of 38℃, which also accelerated mesodermal differentiation, and found that SkM cells exhibited fibre‐like morphology. Finally, after removal of the residual human ESCs by pluripotent stem cell‐targeting delivery of cytotoxic compound, we generated SkM cells with 80% MHC positivity and responsiveness to electrical stimulation. This simple method for myogenic differentiation was applicable to human‐induced pluripotent stem cells and will be beneficial for investigations of disease mechanisms and drug discovery in the future.

## 1 INTRODUCTION

Skeletal muscle (SkM) supports the skeletal system and provides movement to everyday life and tasks; other vital roles include breathing, regulation of body temperature and defecation. Primary culture of human SkM cells is one of the important resources for clarifying human‐specific development and pathomechanisms, although access to this resource is limited. In the last decades, the advent of human embryonic stem cells (ESCs) provided a human resource with unlimited self‐renewal and the ability to differentiate into any type of somatic cells.^1^

Many methods to differentiate human pluripotent stem cells, including human ESCs‐ and human‐induced pluripotent stem cells (human iPSCs), into SkM cells have been established, and ESC‐originated SkM cells have become a potential tool to investigate the mechanisms of development and disease in SkM (Chal and Pourquiél, 2017)^2^. To differentiate human ESCs or iPSCs into SkM cells, there are two major approaches: directed differentiation and direct conversion. The directed‐differentiation approach recapitulates physiological steps in human embryonic development by sequentially adding small molecules or cytokines during weeks of cultivating duration, and it is appropriate for research of the development of infantile‐onset disorders (Chal and Pourquiél, 2017). Conversely, the direct‐conversion approach is achieved by the overexpression of myogenic transcriptional factors (TFs) such as myoblast determination protein 1 (MyoD1), paired box gene 3 (PAX3) and paired box gene 7 (PAX7) (Chal and Pourquiél, 2017). The first idea of the direct‐conversion system was demonstrated by converting fibroblasts to a muscle fate via increased MyoD1,^3^ whose locus is a target of a demethylating agent (5‐azacytidine).^4^ The drug‐inducible expression system, which induces the high expression of MyoD1, offers rapid and robust differentiation methods and opens the way to model SkM‐relevant disorders such as Duchenne or Miyoshi muscular dystrophies.^5^, ^6^, ^7^, ^8^ To deliver myogenic TFs into human pluripotent stem cells for direct conversion, most of the studies described above employed viral or transposon vectors, which cannot avoid the risk of random integration into the host genome.

In this study, we utilized Sendai virus (SeV) vector to deliver Myod1 into human ESCs and iPSCs for direct conversion. SeV is classified as a non‐segmented negative‐strand RNA virus belonging to the *Paramyxoviridae* family. SeV is a cytoplasmic virus,^9^ and it has been developed as a gene‐delivery tool without genomic integration. Induction of TFs via SeV vector has been demonstrated to convert somatic cells into human iPSCs^10^, ^11^ or convert human iPSCs into functional motor neurons.^12^ Therefore, we selected temperature sensitive SeV vector for the Myod1‐delivery vector without risks of genomic integration. Here, we transfected temperature sensitive SeV vector, which encodes *Myod1* (SeV‐Myod1) and converts human ESCs and iPSCs into SkM cells. Additionally, cultivation at a temperature higher than 38℃ can eliminate SeV vector from cytosol^13^ and enhance the differentiation status unexpectedly. Finally, we eliminated the residual undifferentiated human ESCs and iPSCs by pluripotent stem cell‐targeting delivery of cytotoxic compound, and we established a myogenic differentiation method system by using SeV vector encoding Myod1 as a future platform for disease modelling and drug discovery.

## 2 MATERIALS AND METHODS

### 2.1 Generation of SeV18+mMyoD1/TS7∆F

An insert sequence containing the open reading frames of mouse Myod1 (mMyod1) gene was constructed by PCR from cDNAs using NotI‐tagged gene‐specific forward and reverse primers containing SeV‐specific transcriptional regulatory signal sequences. Amplified fragments were inserted into the 18+ region of the plasmid containing SeV/TS7ΔF vector sequence, producing pSeV18+mMyod1/TS7∆F. Recovery and propagation of SeV18+mMyod1/TS7∆F were performed as follows. First, 293T cells were transfected with pSeV18+mMyod1/TS7∆F and pCAGGS plasmids carrying the T7 RNA polymerase and NP, P, F5R and L genes. Cells were maintained in DMEM supplemented with 10% heat‐inactivated foetal bovine serum (FBS) and cultured for 1–3 days to generate seeds of SeV18+mMyod1/TS7∆F vector. The seeds were cloned and propagated using SeV F‐expressing LLC‐MK2/F7/A cells^13^ in MEM containing trypsin (2.5 μg/ml). The titre (cell infectious units/ml) of the recovered SeV18+mMyod1/TS7∆F vector (SeV‐Myod1) was determined by immunostaining method using anti‐SeV rabbit polyclonal serum as described previously.^14^

### 2.2 Myogenic differentiation

H9 human ESCs were obtained from the WiCell Research Institute, Madison, WI. The human ESCs were maintained on 6‐well plates in feeder‐free condition coated with recombinant laminin‐511 E8 fragments (iMatrix‐511; Nippi Inc., Tokyo, Japan) in StemFit medium (AK02N; Ajinomoto, Tokyo, Japan). Human iPSCs, established from healthy individuals,^15^ were also maintained on iMatrix‐511 coating with AK02N medium. On day 0 for SkM differentiation, human ESCs and iPSCs colonies were dissociated into single cells with TrypLE Select enzyme (Gibco; Thermo Fisher Scientific, Inc., Waltham, MA) and seeded onto matrigel‐coated 96‐well plates (#353075, Falcon; Corning, NY) at a cell density of 3 x 10^4^ cells/cm^2^. Cells were infected with SeV‐Myod1 (SeV18+MyoD1/TS7ΔF’, ID Pharma Co., Ltd., Tokyo, Japan) at MOI64 during seeding and again 48 h later. Cells were maintained in αMEM (Nacalai tesque, Kyoto, Japan) supplemented with 5% KSR (Gibco; Thermo Fisher Scientific) and 5 µM Y‐27632 (Nacalai tesque). Medium was changed every other day.

### 2.3 Temperature shift treatment

Seventy‐two hours post‐infection, each individual 96‐well plate was transferred into different incubators (IP400; Yamato Scientific Co., Ltd.) with temperatures set at 37℃, 38℃, 39℃ and 40℃. Cultures at different temperatures were continued until day 8.

### 2.4 Selective elimination of human ESC and iPSC

StemSure® hPSC Remover 50 ng/ml rBC2LCN‐PE38 (#199‐18511; Fujifilm Wako Pure Chemical Corp.) was added to the medium on day 5 and continued for the following 48 h.

### 2.5 Immunofluorescence

On day 8, cells were washed twice with PBS prior to being fixed in 4% paraformaldehyde (#09154‐85; Nacalai tesque) in PBS for 30 min at room temperature and then washed twice with PBS before being blocked with Blocking One histo (#06349‐64; Nacalai tesque) for 30 min at room temperature. Cells were then stained with primary antibodies at 4℃ overnight. Cells were then washed twice with PBS, followed by staining with corresponding fluorescently labelled secondary antibodies or rBC2LCN‐635 (Excitation =634 nm, Emission =654 nm, #185‐03161, Fujifilm Wako Pure Chemical Corp.,) and 4’,6‐diamidino‐2‐phenylindole: DAPI (D9542; Sigma‐Aldrich Corporation) for 2 h at room temperature. The following antibodies were used for immunostaining of dystrophin (ab15277, 1:500; Abcam) ‐myosin heavy chain: MHC (MAB4470, 1:500; R&D Systems, Inc., Mineapolis, MN), myoblast determination protein 1: MyoD (G‐1) (SC377460, 1:500; SantaCruz Biotechnology Inc.), myogenin (5FD) (SC52903, 1:500; Santa Cruz Biotechnology), SeV (MBLPD029, 1:500; Medical and Biological Laboratories Co, Ltd., Woburn, MA), goat anti‐mouse IgG (H+L) Alexa Fluor 488 conjugate (A11029, 1:1,000; LifeTechnologies, Thermo Fisher Scientific) and donkey anti‐rabbit IgG (H+L) Alexa Fluor 555 conjugate (A31572, 1:1,000; Invitrogen, Thermo Fisher Scientific) (Table S1).

### 2.6 Quantitative RT‐PCR

Total RNA was isolated and then reverse‐transcribed by using the SuperPrep® Cell Lysis & RT Kit for qPCR (SCQ‐101; Toyobo Co., Ltd.) on day 8. Quantitative RT‐PCR for muscle‐related genes was performed with SYBR® Premix Ex TaqTM II (Tli RNaseH Plus) (#RR820Q; Takara Bio Inc.,) according to the manufacturer's instructions. Primer information can be found in Table S2.

### 2.7 Assay for cell damage and cell survival

We evaluated cell damage and cell survival 48 h after the infection of SeV vectors. For quantification of cell damage, we collected the supernatant and measured the signal counts of adenylate kinase (ToxiLight bioassay kit; Lonza, Basel, Switzerland) that originated from damaged cells. For quantification of cell survival, we used the commercially available WST‐8 kit (Cell Counting Reagents; Nacalai tesque) and measured the absorbance at 450 nm. We used the condition of 48‐hour incubation with 1 μM staurosporine (SantaCruz Biotechnology) and 1‐h incubation with 1% Triton‐X (Nacalai tesque).

### 2.8 Calcium dynamics analysis to evaluate the contraction of skeletal muscle cells after electrical simulation

iPSC‐derived SkM cells were differentiated in 96‐well plates (black‐wall, flat‐bottom; Greiner Bio‐One). On day 14, SkM cells were loaded for 30 min with 2 μM calcium indicator dye (Fluo‐8AM; AAT Bioquest) and 0.005% Pluronic F‐127 (Sigma‐Aldrich) in Hank's balanced salt buffer without calcium (Nacalai tesque). After 30 min equilibration time, changes in calcium‐dependent intracellular fluorescence, triggered by electric field stimulation (EFS), were measured with a kinetic fluorometric plate reader (FDSS/μCELL; Hamamatsu Photonics, Hamamatsu, Japan). After the electrical field stimulation (voltage 10–20 V, pulse width 4 msec, number of train 2–10 times at a frequency of 2–50 Hz), fluorescence change peak (excitation and emission wavelengths, 480 and 540 nm, respectively) was recorded continuously in the rate of 10 fps for min. Results were expressed as the ratio (ΔF/F) of fluorescence change (ΔF) as compared with the basal level (F).

### 2.9 Statistical analysis

Immunofluorescence data were evaluated using IN Cell Analyzer 6000 (GE Healthcare Life Sciences, Chicago, IL) with the protocol designed by BioInsite (GE Healthcare Life Sciences). All statistical calculations were performed using GraphPad Prism version 7.00 for Windows (GraphPad Software inc., San Diego, CA). Results were analysed by one‐way analysis of variance followed by Tukey's post hoc multiple comparison to determine group differences among different conditions. *p* value <0.05 was considered to show statistical significance.

## 3 RESULTS

### 3.1 Optimized infection of SeV‐Myod1 converted human ESCs into SkM cells

**FIGURE 1:**
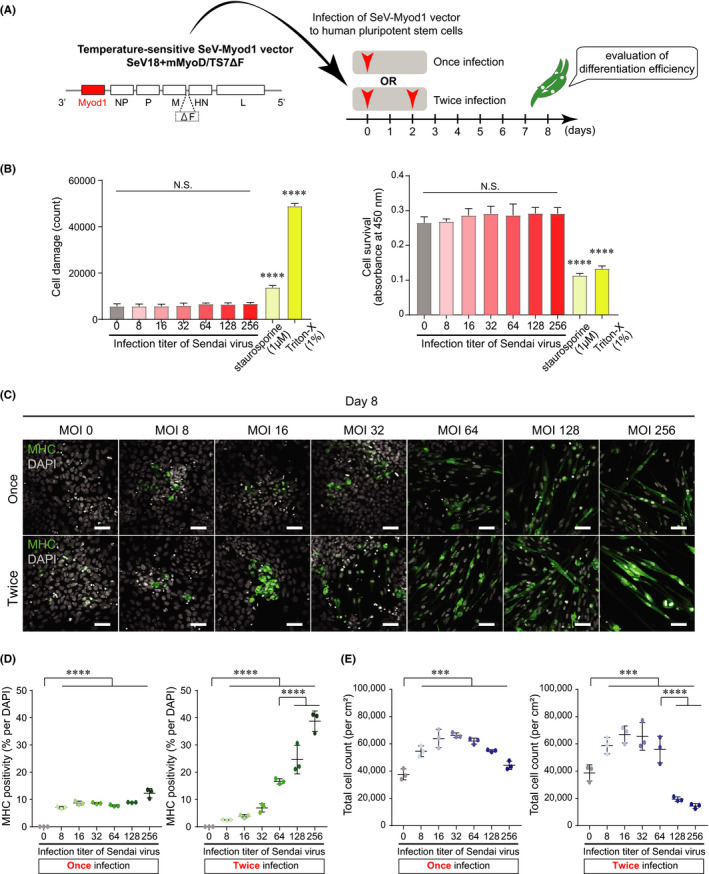
Infection of SeV‐Myod1vector converted human pluripotent stem cells into skeletal muscle cells. (A) Construction of SeV‐Myod1 vector and schema of experimental design. Differentiation efficiency was analysed on day 8 to optimize the SeV‐Myod1 concentration and evaluate the cellular toxicity. (B) Cell damage (left panel) and cell survival (right panel) were analysed at each described MOI condition or at the condition of adding staurosporine and Triton‐X as positive control for cell death. (Independent experiments *n* = 3, mean ± SD, ****p < 0.0001). (C) Immunofluorescence staining for myosin heavy chain (MHC, green) and DAPI staining (white) on day 8. Representative images are shown. Scale bar =100 μm. (D) MHC positivity (% against DAPI) was quantified from (C). Quantified positivity was shown in separate graphs for once infection (left panel) and twice infection (right panel) of SeV‐Myod1. (Independent experiments *n* = 3, mean ± SD, **** *p* < 0.0001) MHC: myosin heavy chain, (E) Total cell number was counted from (C). (Independent experiments *n* = 3, mean ± SD, ***p < 0.001, ****p < 0.0001)

Temperature‐sensitive F‐deficient SeV‐Myod1 was constructed (Figure 1A). Human ESCs were infected with SeV‐Myod1 at different multiplicities of infection (MOI) from 0 to 256 on day 0. To assess the direct toxicity of SeV‐Myod1 on human ESCs, we measured the adenylate kinase released from damaged cells, and we conducted the WST assay to estimate the surviving cells at 24 h after the viral infection (Figure 1B). This assessment of viral toxicity showed that SeV‐Myod1 caused no significant toxicity even at the extremely high multiplicity of infection (MOI 256) (Figure 1B). To convert human ESCs into SkM cells, we added SeV‐Myod1 to human ESCs once on day 0 or twice on days 0 and 2 (Figure 1A), and we compared the positivity of myosin heavy chain (MHC), one of the SkM markers, among different conditions of SeV‐Myod1 infection (Figure 1C). The differentiation efficiency of twice infection was higher than that of once infection especially in the high MOI of 64 to 256 (Figure 1D). However, twice infection at MOI 128 and 256 showed a tremendous decrease in surviving cells on day 8 (Figure 1E). This decrease in surviving cells was due to cell death and/or fused SkM cells, mainly observed in the condition of high MOI (Figure 1C). Cells under the condition of twice SeV‐Myod1 infection at MOI 64 represented around 20% positivity of MHC (Figure 1D) and elongated spindle‐like morphology (Figure 1C) without a massive decrease in surviving cells (Figure 1E). To investigate the background of higher MHC‐positive rate when twice infection was applied, we investigated the time‐dependent changes in the expression of pluripotency markers overtime after SeV‐Myod1 infection and found that the expression level of POU domain, class 5, transcription factor 1 (POU5F1) and NANOG at 48 h after SeV infection was decreased compared with the level before infection (Figure S1A). Furthermore, we compared the different MHC positivities among the different intervals between the first and second infections of SeV‐Myod1 and found that twice infection with a 48‐h interval showed the highest MHC positivity (Figure S1B). Cellular conditions such as early stage of mesoderm‐lineage differentiation and decreased level of pluripotency are known to accelerate myogenic differentiation via MyoD induction,^16^, ^17^ and our SeV‐Myod1 infection may follow this timeline. From these results, twice MOI 64 infection with a 48‐h interval was determined as an optimized condition for the SeV‐Myod1 infection for myogenic differentiation.

### 3.2 Removal of SeV‐Myod1 after cultivation at high temperature

**FIGURE 2:**
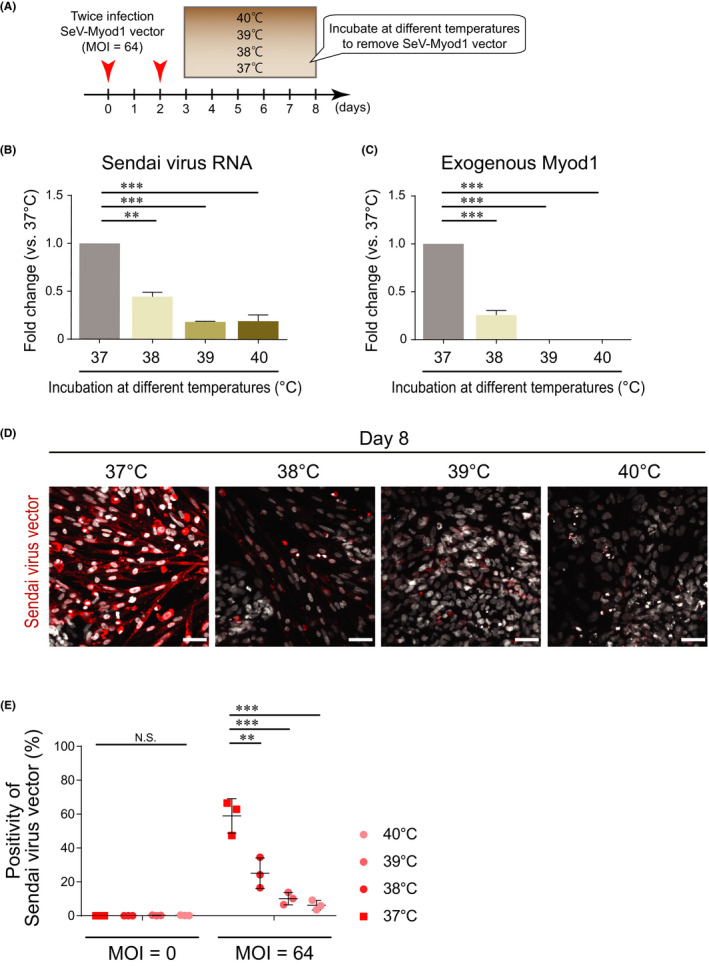
Transient cultivation at high temperature removed infected SeV‐Myod1. (A) Schema of SeV‐Myod1 vector infection and culture condition. The cells were incubated at 37ºC, 38ºC, 39ºC or 40ºC from day 3 to day 8, and the removal of SeV‐Myod1 vector was evaluated. (B) SeV RNA was analysed on day 8 and compared among the different temperature conditions. (Independent experiments *n* = 3, mean ± SD, **p < 0.01, ***p < 0.001). (C) The mRNA level of exogenous Myod1 delivered by SeV‐Myod1 was analysed on day 8 and compared among the different temperature conditions. (Independent experiments *n* = 3, mean ± SD, ***p < 0.001). (D) Differentiating cells were immunostained for SeV antibody (red). Nuclei were stained with DAPI (white). Scale bar = 100 μm. (E) Positivity of Sendai virus vector against total DAPI (%) was quantified and compared among the different temperature conditions. (Independent experiments, *n* = 3, mean ± SD, **p < 0.01, ***p < 0.001, NS: not significant)

The SeV vector used in this study has temperature‐sensitive mutations, meaning that vectors could be removed after incubation at more than 38°C.^11^ To maximize the efficiency of myogenic differentiation by exogenous Myod1, a transient expression was reported to be beneficial.^16^ Therefore, we planned to remove SeV‐Myod1 by utilizing the temperature‐sensitive character of the vector. After twice infection of SeV‐Myod1 at MOI 64, the differentiating cells were cultivated at the higher temperatures of 37°, 38°, 39° or 40℃, compared with the usual conditions for mammalian cells from days 3 to 8 (Figure 2A). We used incubators with a temperature control accuracy of ± 0.1℃. Four incubators had been dedicated to keep different temperatures, 37/38/39/40℃ and to minimize the frequency for door open or door closed. To estimate the removal efficacy of infected SeV‐Myod1 on day 8, we measured the residual RNA of SeV vector (Figure 2B) and exogenous Myod1, which contains a mouse‐specific sequence (Figure 2C). Compared with the 37℃ condition, 5 days cultivation at 38°, 39° and 40℃ significantly removed SeV‐Myod1 vector and exogenous Myod1 (Figure 2B and C). A similar tendency was also confirmed by immunostaining of SeV particles in the cytosolic space of the differentiating cells (Figure 2D, E). These results showed that the SeV‐Myod1 vector can be eliminated by exposure to high temperature.

### 3.3 Cultivation at a higher temperature of 38℃ boosted myogenic differentiation

**FIGURE 3:**
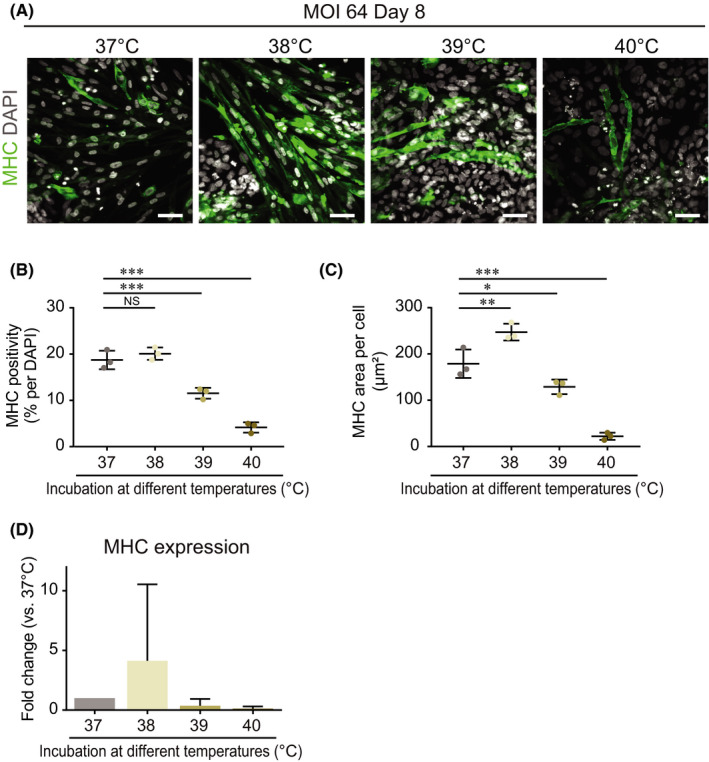
Transient heat shock at temperature of 38ºC accelerated myogenic differentiation. (A) Cells were infected twice with SeV‐Myod1 at MOI 64 and then incubated at 37ºC, 38ºC, 39ºC or 40ºC from day 3 to day 8, as shown in the schema in Figure 2A. Myogenic differentiation efficiency was analysed by immunostaining for myosin heavy chain (MHC, green). Nuclei were stained with DAPI (white) to count the total cell number. Scale bar = 100 μm. (B) MHC positivity (%) against total DAPI was quantified and compared among the different temperature conditions. (Independent experiments *n* = 3, mean ± SD, ***p < 0.001, NS: not significant). (C) MHC‐positive cell area per MHC‐positive cell was quantified and compared among the different temperature conditions. (Independent experiments *n* = 3, mean ±SD, *p < 0.05, ** *p* < 0.01, ***p < 0.001). (D) MHC expression levels were analysed and compared among the different temperature conditions. (Independent experiments *n* = 2, mean ± SD)

We next examined the myogenic differentiation efficiency after exposure to different temperatures (Figure 3A). The population of MHC‐positive cells was 18.7% at the condition of 37℃ and 20.1% at 38℃ (Figure 3B). However, the positivity of MHC was significantly decreased to 11.5% at 39℃ and 4.2% at 40℃ after exposure to higher temperatures (Figure 3A, B). When considering the cell shape on day 8, muscle progenitor cells at the condition of 38℃ had a spindle‐like shape like muscle fibre (Figure 3A) and a larger size in MHC‐positive cells (Figure 3C) compared with the other conditions. In addition, muscle progenitor cells after incubation at 38℃ showed a tendency of higher expression in muscle markers, including MHC (Figure 3D). Furthermore, we investigated the differentiation status of ESCs in the SkM‐differentiation medium without SeV‐Myod1 at 37° or 38℃ and evaluated the effect of the 38℃ treatment on the differentiation propensity. We compared the positivity of differentiation markers for SOX17, α smooth muscle actin (αSMA) and βIII‐tubulin (βIII‐tub) to estimate the differentiation propensity for endoderm, mesoderm and ectoderm, respectively (Figure S2). We also checked the positivity of myosin heavy chain (MHC) to evaluate the direct effect of 38℃ treatment on SkM differentiation of human ESCs. There was no change in the differentiation propensity to ectoderm or endoderm between the 37° and 38℃ culture conditions, but the positivity of αSMA was greatly increased when cultivated in the 38℃ culture condition. These results indicated that 38℃ treatment accelerated mesodermal differentiation from human ESCs and also may enhance the differentiation efficiency into SkM cells in the SeV‐Myod1 system. Taken together, transient heat shock at a temperature of 38℃ was suitable for eliminating SeV vectors and also accelerating myogenic differentiation.

### 3.4 Non‐differentiating pluripotent‐state cells were eliminated with pluripotent cell‐specific killer compound rBC2LCN‐PE38

**FIGURE 4:**
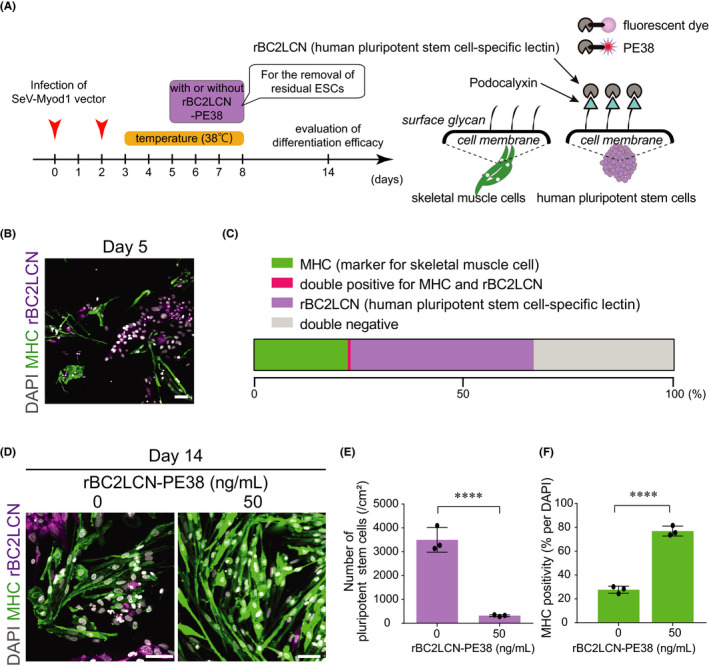
Removal of residual pluripotent stem cells by rBC2LCN‐PE38 enhanced the purity of differentiated skeletal muscle cells. (A) Schema of experimental design. Pluripotent cell killer compound (rBC2LCN‐PE38) was added from day 5 to day 8, and myogenic cell differentiation efficiency was evaluated on day 14. rBC2KCN, a human pluripotent stem cell‐specific lectin, targets the surface of human pluripotent stem cells and is utilized for delivering fluorescent dye or PE38 to human pluripotent stem cells. (B) Presence of pluripotent stem cells on day 5. MHC immunostaining (green) for myogenic cells, rBC2LCN‐fluorescent dye staining (magenta) for pluripotent‐state cell surface representing podocalyxin. Nuclei were stained with DAPI (white). Scale bar = 100 μm. (C) Stacked bar charts of the percentage of MHC‐positive cells and human ESCs stained by rBC2LCN‐635. There was almost no overlap between MHC‐ and rBC2LCN‐positive cells on day 5. (D) Residual human ESCs on day 14 were stained with MHC antibody or rBC2LCN‐635 dye and compared between groups without (0 ng/ml) or with (50 ng/ml) rBC2LCN‐PE38. Scale bar = 100 μm. (E) Numbers of rBC2LCN‐fluorescent dye‐stained pluripotent stem cells were compared after treatment without or with rBC2LCN‐PE38 (50 ng/ml). (Independent experiments, *n* = 3, mean ± SD, ****p < 0.0001). (F) MHC positivity (%) was compared after treatment without or with rBC2LCN‐PE38 (50 ng/ml). (Independent experiments, *n* = 3, mean ± SD, ****p < 0.0001)

We converted human ESCs into SkM cells by simply adding SeV‐Myod1 and a heat exposure of 38℃. However, the differentiation efficiency remained around 20% due to the residual human ESCs, which had not been infected by SeV‐Myod1 vectors. These residual human ESCs will proliferate massively and cause a decrease in differentiation efficiency to SkM cells. To eliminate the human ESCs in a dish, we utilized a human pluripotent stem cell‐specific lectin^18^ to deliver the 38 kDa domain of exotoxin (PE38) to human ESCs (Figure 4A). rBC2LCN, a lectin designated recombinant N‐terminal domain of BC2L‐C lectin derived from *Burkholderia cenocepacia* (rBC2LCN), can label human ESCs and iPSCs with fluorescent dye, and rBC2LCN‐PE38 is designed to selectively remove undifferentiated pluripotent‐state cells.^19^, ^20^ At first, we evaluated the positivity of MHC and rBC2LCN (Figure 4B) and found that there was almost no overlap between their positive staining on day 5 (Figure 4C). Taking advantage of this character, we added rBC2LCN conjugated with PE38 from days 5 to 8 to eliminate the residual human ESCs that were positive for rBC2LCN. On day 14, rBC2LCN‐PE38 removed almost all of the residual human ESCs (Figure 4D, E) and enhanced the efficiency of myogenic differentiation from 27.6% to 76.9% (Figure 4F). There were also a small number of MHC‐negative cells without pluripotency marker (Figure S3A‐D). These double‐negative cells showed small‐round or flattened proportion (Figure S3E‐H), which may be resistant to conversion to SkM cells after SeV‐Myod1 vector infection and escape from the cytotoxicity of rBC2LCN‐PE38. We also confirmed the removal of SeV‐Myod1 vector on day 14 after the 38℃ treatment (Figure S4A), and that SkM cells differentiated by the established method on day 14 expressed the SkM‐related genes (Figure S4B). These results illustrate that we established the myogenic differentiation method by SeV‐Myod1 infection following transient heat shock and removal of residual human ESCs.

### 3.5 Application of the established method to human iPSC

**FIGURE 5:**
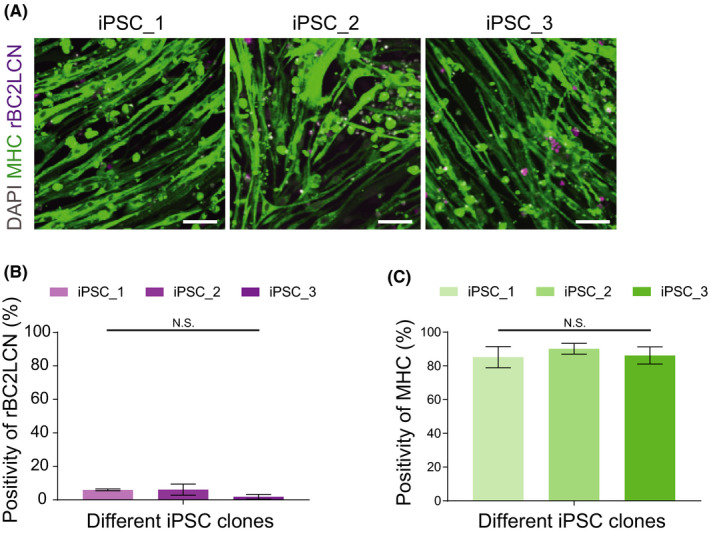
SeV‐Myod1 and 38℃ treatment converted multiple induced pluripotent stem cells (iPSCs) into skeletal muscle (SkM) cells. (A) Immunostaining of SkM cells originating from three different iPSCs were immunostained by myosin heavy chain (MHC) antibody (green), rBC2LCN‐635 dye (magenta) and nucleus‐stained with DAPI (grey). Scale bars = 100 μm. (B) Positivities of rBC2LCN were calculated. Independent experiment, *n* = 3, mean ± S.D. (C) Positivities of MHC were calculated. Independent experiment, *n* = 3, mean ± S.D

We applied the established method to human iPSCs, which is a powerful tool for disease modelling and further drug investigations. We utilized three different iPSC clones that originated from healthy individuals.^15^ As results similar to those with human ESCs, the infection by SeV‐Myod1 followed by the 38℃ condition and rBC2LCN‐PE38 treatment successfully converted all three human iPSCs into SkM cells with a similar efficiency to the case of human ESCs (Figure 5A‐C). These results showed that the established differentiation method for SkM cells can be applied to human iPSCs.

### 3.6 Differentiated SkM cells responded to electrical stimulation

**FIGURE 6:**
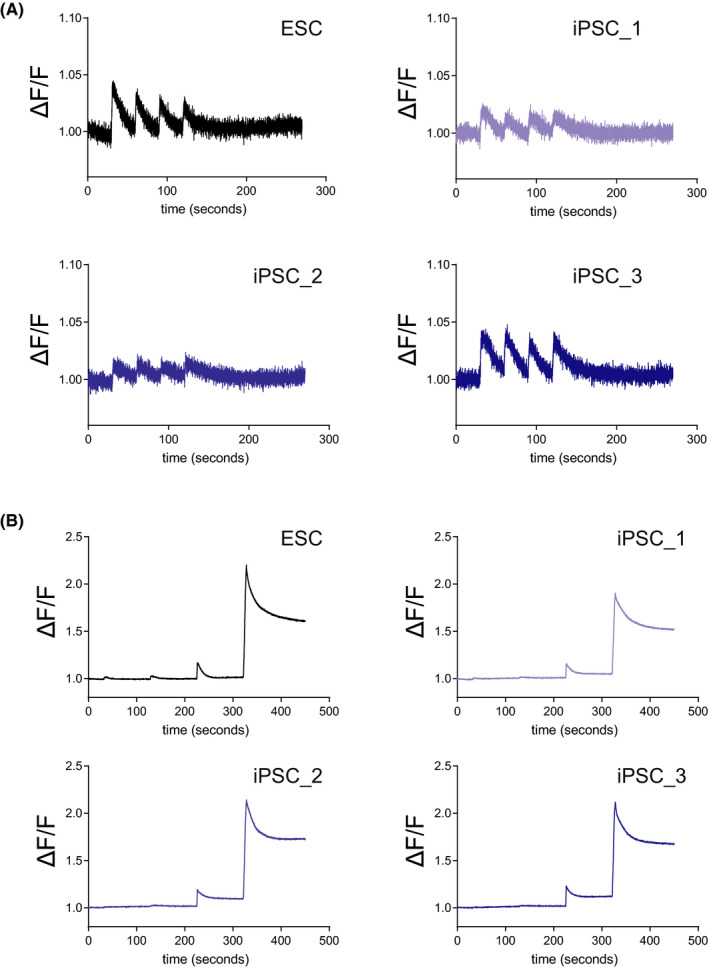
SkM cells responded to electrical stimulations. (A) Differentiated SkM cells were cultured in a 96‐well plate. To evoke the Ca^2+^ response, SkM cells were subjected to 3 Hz electrical field stimulation (EFS) and were monitored by analysing the altered fluorescence, indicating the altered dynamics of cytosolic Ca^2+^ concentration, quantified as ΔF/F. (B) ΔF/F were monitored after EFS at 10 V/2 Hz, 10 V/10 Hz, 10 V/50 Hz and 20 V/50 Hz sequentially at 90‐second intervals

To confirm the functions of the differentiated SkM cells from human ESCs and iPSCs, we used electrical stimulation on human ESC‐ and iPSC‐derived SkM cells and investigated the functional activity by monitoring the intracellular calcium concentration. Calcium plays a key role in regulating the SkM contraction, and monitoring of calcium dynamics can provide functional information of in vitro SkM cells.^21^ To construct the assay system, we used the calcium transients that were reported to coincide with twitch and tetanus responses, and that the alteration in the intensity of the calcium indicator, quantified as ΔF/F, was proportional to the magnitude of the applied force.^22^ We monitored the time‐dependent alteration in fluorescent intensity of the calcium indicator, described as ΔF/F, after adding electric field stimulation (EFS). At first, we used single stimulation, 4‐msec pulse width of 10 V, 3 Hz and found the active Ca^2+^ influx at the same timing as electrical stimulation (Figure 6A). Next, we also used high‐frequency stimulation, a 4‐msec pulse width of 10 – 20 V, 2–50 Hz, and found that the high‐frequency stimulation could evoke a drastically higher ΔF/F (Figure 6B), which is widely observed in the tetanic contraction of primary SkM cells or in in vivo analysis. From these results, we also discussed the importance of functional assays and future perspectives of co‐culture with motor neurons from human iPSCs to further investigate physiological assays.

## 4 DISCUSSION

In this study, we established a simple and efficient method to generate SkM cells from human ESCs and iPSCs using SeV encoding Myod1. This method enables us to obtain a high yield of SkM cells without complicated cell culture processes in directed‐differentiation methods using step‐wise addition of various cytokines or small compounds, nor with direct‐conversion methods that reacquire cell isolation steps and/or sub‐cloning steps of human iPSCs.^23^

Exogenous Myod1 gene induction is achieved by simply adding SeV‐Myod1 to the culture medium, and no other genetic manipulation is required (Figure 1A). SeV vector has advantages over other viral vectors because SeV vector consists of cytoplasmic RNA, and they express their genes within the cytoplasm without entering the nucleus. Therefore, the SeV vector has no risk of integration into the host genome, thereby providing a potential therapeutic application. In addition, the SeV vector can be applied to both dividing and non‐dividing cells, and short‐term exposure is enough for efficient transduction. Moreover, SeV vector has been modified to be rapidly eliminated to minimize its effect on the host cells.^9^, ^24^ The established direct‐conversion method using integration‐free SeV vector can be applied for pathological analysis and drug screening assays, which require the use of many different human iPSC lines derived from different patients.

One advantage of our method is the elimination of residual SeV by transient exposure to the cultivation at a treatment temperature of 38℃, as the SeV vector in this study carries a temperature‐sensitive mutation.^11^ Interestingly, treatment at 38℃ increased both the differentiation efficiency and the number of cells with spindle‐like elongated muscle cell morphology. We speculate that this could be due to increased expressions of specific heat shock proteins. A previous study reported that heat shock proteins (HSPs), including MKBP/HSPB2 and HSPB3, are induced during muscle differentiation under the control of Myod1, suggesting that these HSP oligomers might have an additional system closely related to muscle functions.^25^ Others showed that the levels of HSPs, namely HSP25, HSP40, HSP90 and HSP110, were highly elevated in 50% confluent proliferating myoblasts.^26^, ^27^ It has also been shown that HSPs play a crucial role in myogenesis. McArdle et al. showed that HSP70 overexpression facilitates muscle regeneration.^28^ Barone et al. found increased skeletal muscle HSP60 after endurance training.^29^ Taken together, it is suggested that transient exposure at a temperature of 38℃ might act like heat shock and improve myogenic differentiation. For further disease modelling of motor neuron diseases, we previously reported the generation of motor neurons using SeV encoding three transcription factors: LIM/homeobox protein 3, neurogenin 2 and islet‐1.^12^ By combining the SeV‐based differentiation system into motor neurons or SkM cells from patient iPSCs with various disorders in the neuro‐skeletal muscle system, we can open the way for disease modelling and drug discovery in the future.

## CONFLICT OF INTEREST

Jitsutaro Kawaguchi and Tsugumine Shu are employees of I'rom Group Co., Ltd. The remaining authors declare no competing interests.

## AUTHOR CONTRIBUTIONS

**Ghee Wan Tan:** Data curation (equal); Formal analysis (equal); Validation (equal); Writing‐original draft (equal). **Takayuki Kondo:** Conceptualization (equal); Investigation (equal); Resources (equal); Validation (equal); Visualization (equal); Writing‐original draft (equal); Writing‐review & editing (equal). **Keiko Imamura:** Supervision (supporting). **Mika Suga:** Supervision (supporting); Writing‐original draft (supporting). **Takako Enami:** Resources (supporting). **Ayako Nagahashi:** Visualization (supporting). **Kayoko Tsukita:** Validation (supporting). **Jitsutaro Kawaguchi:** Resources (equal). **Tsugumine Shu:** Resources (equal). **Haruhisa Inoue:** Conceptualization (equal); Investigation (equal); Writing‐review & editing (equal). **Ikuyo Inoue:** Validation (supporting).

## Supporting information

Figure S1‐S4

Table S1

Table S2

Supplementary Material

## Data Availability

The data that support the findings of this study are available from the corresponding author upon reasonable request.
