# Supplementary figures and images for: Simple derivation of skeletal muscle from human pluripotent stem cells using temperature‐sensitive Sendai virus vector
Source: J Cell Mol Med. 2021 Sep 12;25(20):9586–96. doi: 10.1111/jcmm.16899 (PMC8505837; doi:10.1111/jcmm.16899)

# Figure S1

---

A

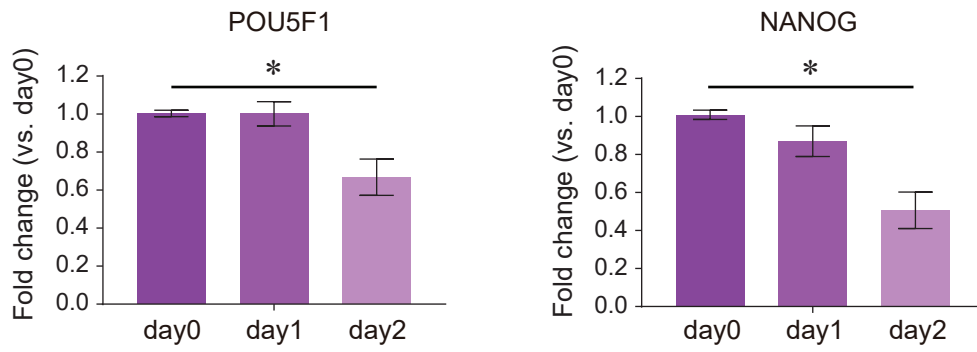

B

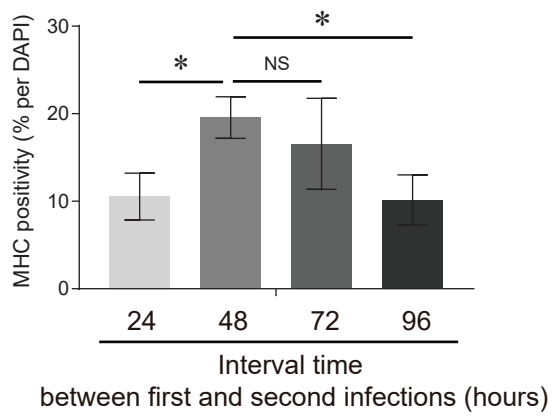

# Figure S2

A

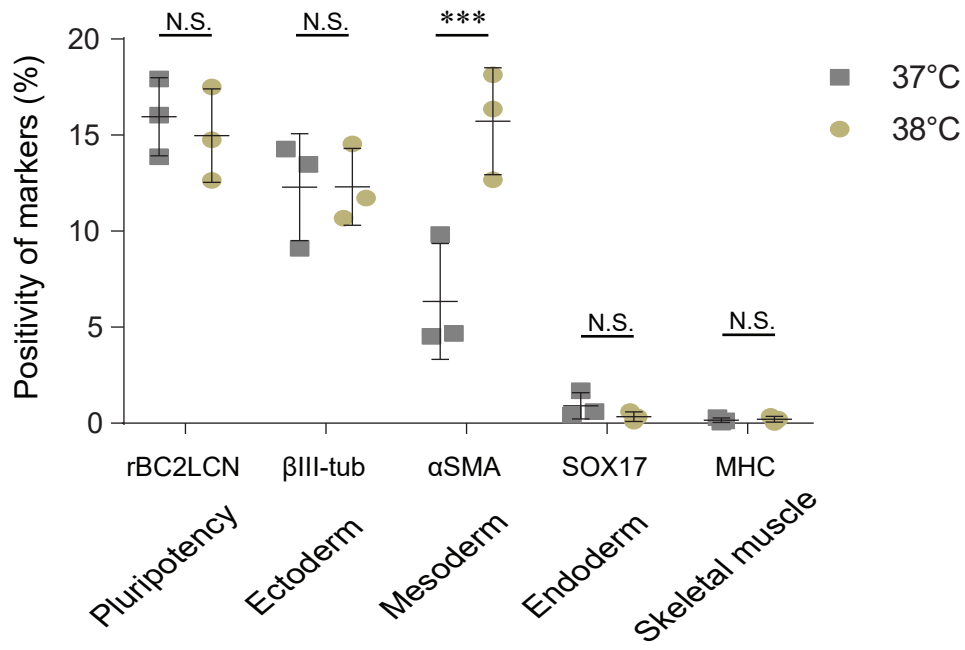

# Figure S3

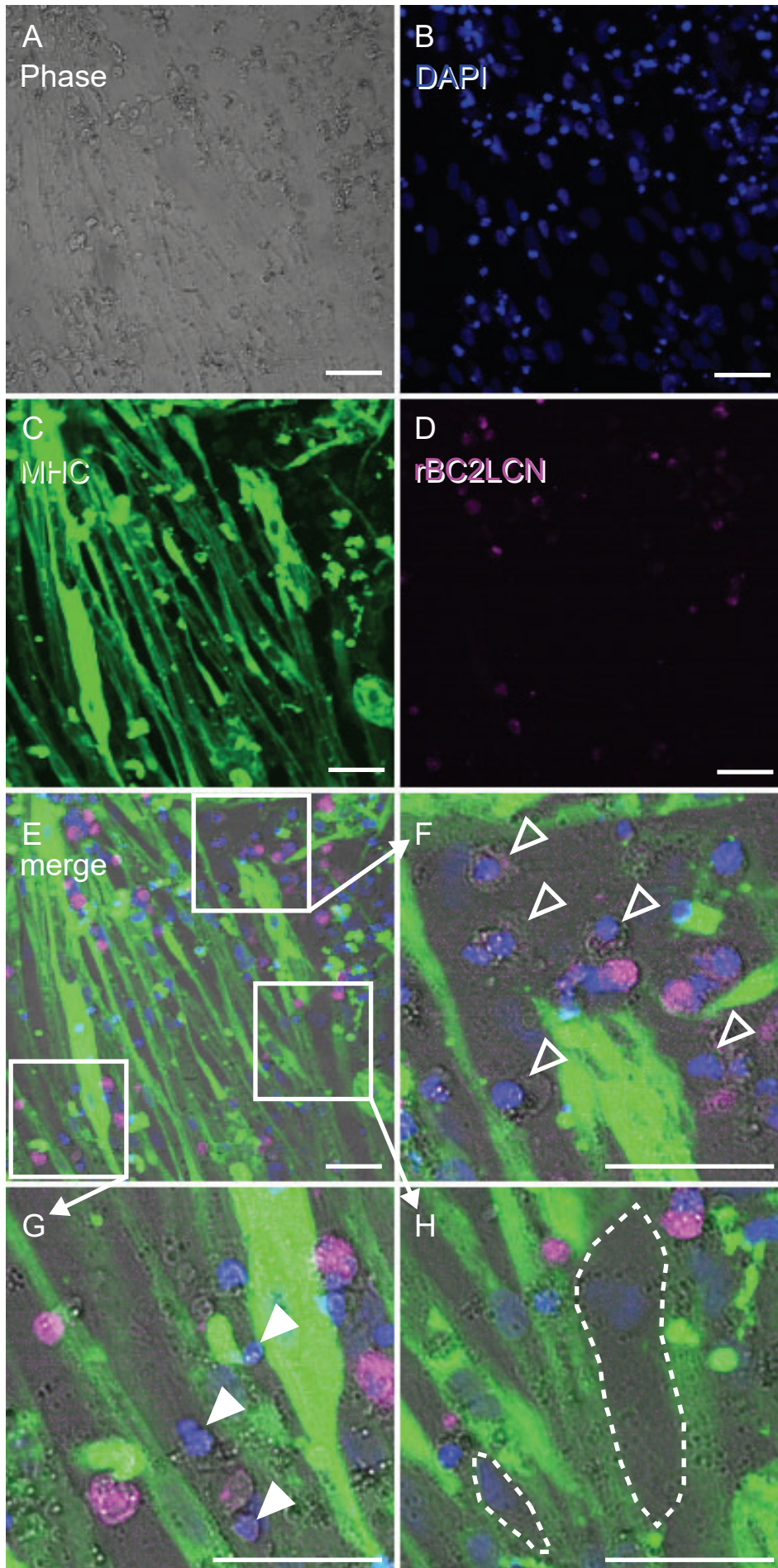

# Figure S4

A

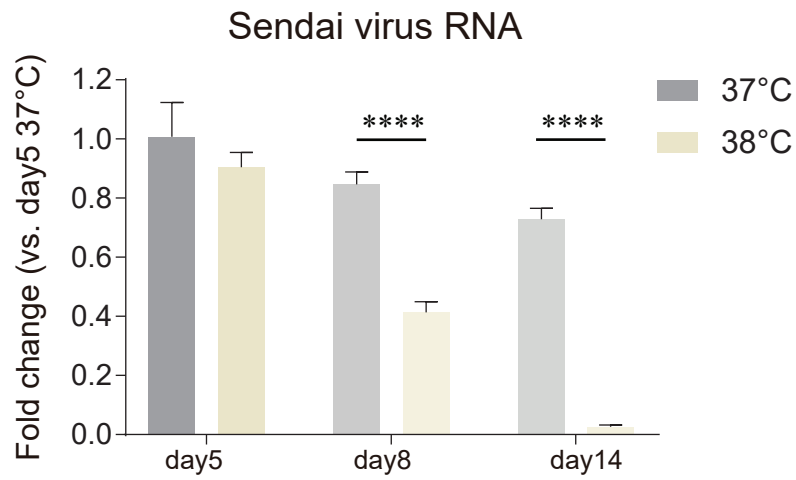

B

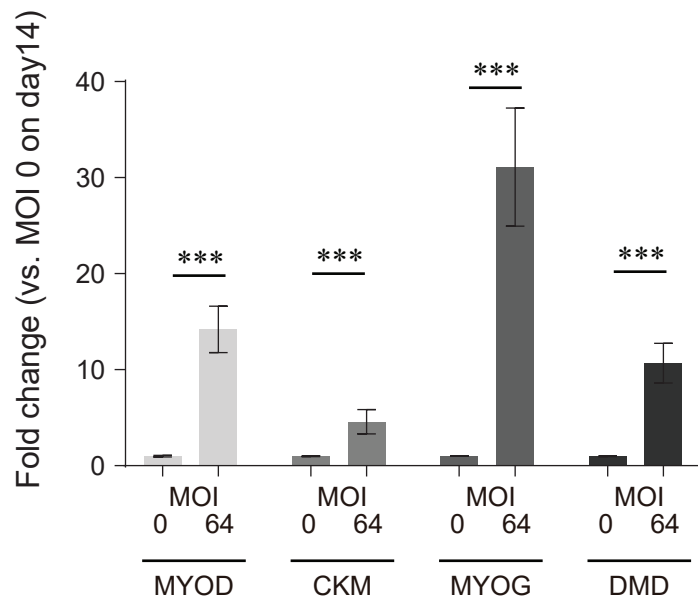

Supplement: Supplementary file 1 — Figure S1‐S4 [file JCMM-25-9586-s001.pdf]
