# Supplementary material for: Simple derivation of skeletal muscle from human pluripotent stem cells using temperature‐sensitive Sendai virus vector
Source: J Cell Mol Med. 2021 Sep 12;25(20):9586–96. doi: 10.1111/jcmm.16899 (PMC8505837; doi:10.1111/jcmm.16899)
Supplement: Supplementary file 2 — Table S1 [file JCMM-25-9586-s004.docx]

**Table S1: Antibodies used for immunocytochemistry**

| Target protein or antibody clone | Dilution | Vendor  Catalogue number  RRID |
| --- | --- | --- |
| Human Myosin Heavy Chain | 1:500 | R&D Systems  Cat# MAB4470  RRID: AB_1293549 |
| Sendai Virus | 1:500 | Medical and Biological Laboratories Co, Ltd.  Cat# PD029  RRID: AB_10597564 |
| βIII tubulin | 1:2,000 | Chemicon  Cat# CBL412  RRID:AB_11205398 |
| alpha-Smooth Muscle Actin (SMA) | 1:1,000 | DAKO  Cat# M0851  RRID: AB_2223500 |
| SOX17 | 1:400 | R&D Systems  Cat#: AF1924-SP  RRID:AB_355060 |
| Goat anti-Mouse IgG (H+L) Alexa Fluor® 488 conjugate | 1:1,000 | LifeTechnologies, Thermo Fisher Scientific  Cat# A11029  RRID: AB_138404 |
| Donkey anti-Rabbit IgG (H+L) Alexa Fluor® 555 conjugate | 1:1,000 | Invitrogen, Thermo Fisher Scientific  Cat# A31572  RRID: AB_162543 |
| Donkey anti-Rabbit IgG (H+L) Alexa Fluor® 555 conjugate | 1:1,000 | Invitrogen, Thermo Fisher Scientific  Cat# A31572  RRID: AB_162543 |
