# Supplementary material for: Simple derivation of skeletal muscle from human pluripotent stem cells using temperature‐sensitive Sendai virus vector
Source: J Cell Mol Med. 2021 Sep 12;25(20):9586–96. doi: 10.1111/jcmm.16899 (PMC8505837; doi:10.1111/jcmm.16899)
Supplement: Supplementary file 3 — Table S2 [file JCMM-25-9586-s003.docx]

**Table S2: Primers used for quantifying mRNA expression by RT-qPCR**

| Target | Forward | Reverse |
| --- | --- | --- |
| *MYH2*  *(MHC)* | AACTTCAGGCAAAAGTGAAATCTT | GCTAGATTGGTGTTGGATTGTTC |
| *Myod1*  (SeV-encoded) | AGCACTACAGTGGCGACTCA | GGCCGCTGTAATCCATCAT |
| Sendai virus | GGATCACTAGGTGATATCGAGC | ACCAGACAAGAGTTTAAGAGATATGTATC |
| *MYOD* | CGCCATCCGCTATATCGAGG | CTGTAGTCCATCATGCCGTCG |
| *CKM* | ACCTCAACCATGAAAACCTCA | GGCTGCTGAGCACGTAGTTA |
| *MYOG* | GCTCAGCTCCCTCAACCA | GCTGTGAGAGCTGCATTCG |
| *DMD* | GATGCTGTTTTGCACTATCTTGA | TTCTTTTGAACATCTTCTCTTTCATC |
| *ACTB* | CCAACCGCGAGAAGATGA | TCCATCACGATGCCAGTG |
