# Supplementary material for: Simple derivation of skeletal muscle from human pluripotent stem cells using temperature‐sensitive Sendai virus vector
Source: J Cell Mol Med. 2021 Sep 12;25(20):9586–96. doi: 10.1111/jcmm.16899 (PMC8505837; doi:10.1111/jcmm.16899)
Supplement: Supplementary file 4 — Supplementary Material [file JCMM-25-9586-s002.docx]

**FIGURE S1**

**Optimizing the timing for the infection of Sendai virus vectors.**

(A) The expression of pluripotency markers, including *POU5F1* and *NANOG*, was analysed on days 0, 1 and 2, and compared among the different days after the first infection of SeV-Myod1. (Independent experiments, n = 3, mean ± SD, * p < 0.05).

(B) MHC positivity against total DAPI (%) was quantified and compared among the different interval 24-96 hours between the first and second infections of SeV-Myod1. (Independent experiments, n = 3, mean ± SD, * p < 0.05, NS: not significant)

**FIGURE S2**

**Evaluating the effect of 38°C treatment on the differentiation propensity of uninfected cells.**

(A) Pluripotency marker (rBC2LCN), ectoderm-lineage marker (βIII-tubulin (βIII-tub)), mesoderm-lineage marker (α smooth muscle actin (αSMA)), endoderm-lineage marker (SOX17) and myosin heavy chain (MHC) positivity against total DAPI (%) without SeV-Myod1 infection on day 14 was quantified and compared between the 37 and 38°C conditions. (Independent experiments, n = 3, mean ± SD, *** p < 0.001, NS: not significant)

**FIGURE S3**

**Cell proportion of MHC-negative and rBC2LCN-negative cells on day 14.**

(A) Phase image, (B) DAPI (blue), (C) MHC (green), (D) rBC2LCN (magenta), (E-H) merged images of ESC-derived SkM cells on day 14. (F-H) show magnified images of insets in (E). Scale bars = 100 μm. (F) Open arrowheads indicate MHC-negative and rBC2LCN-negative cells with small-round shape. (G) Arrowheads indicate MHC-negative and rBC2LCN-negative cells with dense and irregular-bordered nucleus. (H) Dashed lines indicate MHC-negative and rBC2LCN-negative cells with flattened shape and relatively large cytosol.

**FIGURE S4**

**Expression status of remaining SeV or muscle-related genes on day 14.**

(A) The remaining SeV-Myod1 vector was analysed on days 5, 8 and 14, and compared between the different conditions at 37° or 38°C treatment. (Independent experiments, n = 3, mean ± SD, **** p < 0.0001).

(B) The expression of SkM-related markers, including myoblast determination protein 1 (*MYOD), creatine kinase, muscle (CKM), myogenin (MYOG) and Dystrophin (DMD)*, was analysed on day 14 and compared between the conditions with or without SeV-Myod1 infection. (Independent experiments, n = 3, mean ± SD, *** p < 0.001).
